# A Modern Occupation for Women: Feminization and the Professionalization of Nursing in 1930s’ China

**DOI:** 10.1353/tcc.2025.a950427

**Published:** 2025-01-30

**Authors:** Yier Xu

**Affiliations:** Newcastle University

**Keywords:** nursing, gender, medical professionals, the Modern Girl, the New Woman

## Abstract

When nursing was first introduced in China in the late 1800s, it was an occupation to which men and women had equal access. But by the mid 1930s, the ratio between female and male nurses in China was more than two to one. The feminization of nursing during the Republican era was caused by the departure of male nurses who left in pursuit of better wages and was animated by competing discourses centered around the tropes of the Modern Girl and the New Woman. Civilian hospitals facilitated the feminization of nursing by utilizing these gender tropes to their advantage. They justified hiring more women on the grounds that a nurse’s duties resembled a woman’s domestic responsibilities. The hospitals objectified their nursing staff’s femininity to attract patients. And they highlighted the nurses’ image as modern women to imply the modernity of their hospital. When patients criticized the attributes of the Modern Girl assumed by nurses, female nurses asserted their authority over knowledge, gender roles and merit to defend themselves and promote their professional image. These efforts by female nurses to professionalize their work played a key role in the ultimate success of the feminization of nursing.

A survey carried out by the Nursing Association of China (中華護士學會 Zhonghua hushi xuehui, NAC) in 1935, indicates that there were 1,477 male nurses and 3,328 female nurses employed nationwide.^1^ One year later, the same association listed the names of fourteen newly registered members, only two of whom were men.^2^ The low ratio of male nurses was noteworthy because men and women had equal access to nursing education in the previous decade. The nursing school of Renji Hospital (仁濟醫院 *renji yiyuan*), a missionary hospital in Shanghai, for example, admitted ten male and ten female pupils in 1920. The next year, the number of male students admitted exceeded that of their female counterparts.^3^ A missionary-run hospital in Guangxi province also showed that doctors trained both male and female nurses.^4^

In 1909, missionary nurses set up the NAC to standardize the work and training of nurses. The association was available to all nurses in China regardless of their nationality or gender.^5^ The professional association and the Chinese government allowed full access to men in nurse training in the 1920s and 1930s. And when the Nationalist party established its government in Nanjing in 1928, and changed the Public Health Branch of Executive Yuan (行政院衛生司 *Xingzhengyuan weisheng si*) into the Ministry of Public Health Administration (衛生部 *Weisheng bu*) to carrying out the NAC’s regulation of nurse training,^6^ it continued the NAC’s policy and did not prevent men from studying nursing.

Nevertheless, nursing became a women dominated profession by the mid 1930s. This study argues that the feminization of nursing occurred as a response to several cultural and social factors. Low pay drove male nurses away from civilian hospitals; hospitals used the tropes of the Modern Girl and the New Woman to attract patients and boost their revenue; and female nurses redoubled their efforts to professionalize nursing in response to the criticisms levelled against them for embodying attributes of the Modern Girl.

In the 1930s and 1940s, tangled discussions of the Modern Girl and the adapted trope of the New-Woman-responding-to-mass-consumerism, ran parallel to the development of nursing. Beginning with the New Culture Movement, intellectuals promoted the image of the New Woman—an idealized modern women pursuing education, self-selected marriage, and devoted to nation building.^7^ This prototype had a significant impact on women’s identity in the following decade, becoming gradually more controversial in the late 1920s. When consumer culture flourished in the Nanjing decade, the New Woman trope was increasingly featured in advertisements. The “new women” promoted in advertisements was criticized by leftist intellectuals as pseudo-modern – a figure that seemed to pursue a fashionable lifestyle but remained indifferent to political issues or social improvement. The advertisers’ pseudo “new women” were called the Modern Girl by leftist intellectuals…^8^ who considered the women in the images as vain and irresponsible, caring only about entertainment and petty matters.

The Modern Girl was also criticized by the Nationalist government during the New Life Movement begun in 1934. This movement was a state-initiated, conservative movement that countered commercialism and liberalism. The Modern Girl, who had her origin in discourse on women’s liberation and who embodied consumerism, became a target of the Nationalist government in the New Life Movement.^9^ Yet, the New Woman remained popular among women in the 1930s, albeit with new features. As Gail Hershatter has observed, the New Woman at the time was “eager to acquire knowledge and attain economic independence, trained in a profession, not subservient to men, frugal, modest in dress and self-presentation, politically informed, [and] attuned to contemporary social problems.”^10^ The new features that were associated with consumption were developed in response to the consumer culture and the New Life Movement’s emphasis on frugality.

Women’s experience in the workplace, their image in the public sphere, and their self-identification were influenced by opinions about gender roles and feminine virtues.^11^ Historians have demonstrated that women who worked in public settings faced harassment, gender inequality and limited access to leadership, all influenced by conservative opinion about femininity and gender roles.^12^ While gender complicated women’s experience in the workplace, the idea of the New Woman inspired them to resist oppression and to struggle for a better future for themselves.^13^ Although career women were associated with modernity in popular discussion and they themselves identified with the standard of the modern woman, their understanding of their own work was still shaped by the conventional gender roles: housewife and mother. Hershatter’s oral interviews with women in rural China in the Maoist era reveal the persistence of this thinking.^14^

Because gender discourse has had a significant impact on the trajectory of women’s careers, gender has been a focus of historical studies on nursing. Just how gender roles, conventional views of feminine virtues, and popular tropes of women as new or modern, formed the nursing profession in civilian hospitals, however, has not been studied. Furthermore, although the feminization of nursing affected both male and female nurses, the experience of male nurses has been understudied. During the Second Sino-Japanese War, studies on wartime nursing suggest that the labor shortage, the increased mobility of the population, the patriotism of female nurses and their empathy for soldiers and patients in rural areas all contributed to the feminization of nursing in that period.^15^ The professionalization and feminization of nursing, however, began well before the outbreak of war. This article examines nursing in urban-based civilian hospitals in the immediate pre-war period and considers the careers of male nurses. It uses documents from both the central and local governments to understand the dynamic of the institutionalization of nursing in a top-down process. The research for this article examined magazine and newspaper articles published in Shanghai, Nanjing, Guilin, and Nanning to appreciate the cultural construction of nursing and nurses’ views. By studying nursing in both larger, wealthier cities and in smaller ones, I establish that the regulation of medical professionals impacted their career outlook, though to varying degrees in different cities. I also demonstrate that the influence of gender stereotypes and cultural symbols extended beyond the cultural or political centers of Shanghai and Nanjing.

## Career Paths for Male Nurses

The government took the initiative to regulate nursing and nurse training in the 1930s. Before state intervention, nurses and their training were under the supervision of the NAC, a private organization, founded by missionary nurses. The aims of the Association included setting up standardized courses and assuring the quality of nurses’ work. To achieve these goals, members translated Western textbooks and designed courses available to hospitals where their members were based. The association also held examinations for nursing students to assess their qualifications.^16^ In 1930, Liu Ruiheng (劉瑞恆 1890-1961) the Minister of the Public Health Administration, spoke at the tenth national congress of the NAC. There, he advised the committee to facilitate communication between the Public Health Administration and the NAC by moving its headquarters to Nanjing where the central government was based. Following Liu’s speech, the NAC handed over the regulation of nursing to the Administration and, in 1932, the NAC came under the auspices of the government.^17^ The association formed a close relationship with the central government. By 1934, members of the former NAC, Pan Jingzhi (潘景芝 1901-1968), Shi Xien (施锡恩 ?-?), Liu Ganqing (劉幹卿 1900-1972), Chen Zhu Bihui (陳朱碧輝 1901-?) and Hu Dunwu (胡惇五 1898-1974), worked to standardize nurse training for the Nursing Education Committee in the central government (中央護士教育委員會 Zhongyang hushi jiaoyu weiyuanhui).^18^ Throughout the Republican era, the NAC maintained a close relationship with the state, collaborating on regulating nurses, and in turn, reflecting the Nationalist Government’s support for nursing.

State intervention in the regulation of nursing was part of the broader oversight of medical professionals. The GMD government began to regulate doctors in 1929, one year after the establishment of the Public Health Administration. The committee reached a consensus at its first conference: Doctors who practiced Western medicine should be licensed by the government by the end of 1929, and practitioners of traditional Chinese medicine (TCM) should be registered during 1930. The idea here was to exclude from practice those who were not qualified.^19^ To achieve this aim, the central government mandated that doctors pass the state examination before being licensed whether they were trained at registered medical schools or not.^20^ Provincial governments followed suit. In 1935 -- indicating the limited influence of the central government’s mandate up to that point -- Guangxi ordered medical schools, research institutions, county governments, and police offices to clamp down on doctors who practiced Western medicine without a license.^21^ Despite past failures, the new ordinance from Guangxi’s provincial government revealed their determination to regulate the practice of medicine. Ultimately, the ordinance succeeded in preventing many who were not qualified from practicing medicine.

Stricter regulation on doctors influenced the career outlook of male nurses. Before the Nationalist Government issued its regulation on medical professionals, doctors’ qualifications were checked only loosely. The Beiyang Government in Beijing, for example, issued an ordinance on the registration of medical professionals in 1915, but its influence was limited. The police who were responsible for verifying individual doctor’s registration, lacked experience, and only caused conflicts between the authorities and the medical professionals. It was difficult to enforce the law in Beijing, let alone other areas.^22^ As there was not a proper way to assess doctors’ professional qualifications, male nurses found an opening to practice medicine upon graduation from nursing school. Some of them worked as military doctors, and some ran their own clinics.^23^ The regulation put into force by the Nationalist Government from the late 1920s proved effective in stopping unqualified people, such as male nurses, from practicing medicine. As scholarship on the professionalization of the medical industry has shown, regulating doctors evoked debates on quackery between practitioners of different kinds of medicine. Through debates in the press, both the medical profession and the general population gained a better understanding of what it meant to be a qualified doctor.^24^ The general population gradually realized that educational background mattered in terms of doctors’ professional qualifications. In 1936, the Shanghai press reported on a man with a nursing education who practiced as a doctor mistakenly administered a lethal injection.^25^ This highlight on the man’s educational background suggests that the scope of education had become key to the public’s understanding of the professional qualification needed for a medical practitioner.

This change in public opinion and medical practice was also observed in Guilin. In the 1920s, Chinese assistants could practice medicine in missionary hospitals in Guilin, even though they had not received an education in a medical school.^26^ There were loose definitions of the role of nurse or doctor, and distinctions between the two remained murky. In the mid 1930s, the assistants were required to complete a program on medicine at the university, even though they had served in missionary-run hospitals for years.^27^ The distinction between different medical roles became even clearer in regions that lacked medical resources and had less exposure to foreign influence. By at least the mid 1930s, it became more difficult for male nurses to take on doctors’ work.

Despite the growing consensus that there should be a strict division between nurses and doctors, articles in medical journals showed sympathy to the male nurses who worked as doctors. In 1935, an article in *Yishi gonglun* (醫事公論 Public opinions on medicine) reported that male nurses acted as doctors due to the dire prospects they faced in nursing. In the article, Tian Weifan (田維范) said more women were recruited as nurses in response to patient demand, making it more difficult for male nurses to find jobs in civilian hospitals. Tian reasoned that women were gentler by nature and more suitable for care taking roles and he claimed that this was the consensus of the general population.^28^ Tian’s account of the popular view of nursing indicated that the contact between female nurses and male patients was not something people opposed, which created an opportunity for women to enter the nursing field. Females working as nurses in hospitals, however, were subject to an added level of scrutiny based on ascribed gender roles and morality.

Apart from hospitals’ growing preference for female nurses, low pay also drove away men, who found it difficult to work as a nurse and still be the breadwinner for the family. ^29^ Xiao Linlin’s study on nursing in Shanghai demonstrates that nurses’ incomes varied from 8 yuan to 80 yuan per month in 1935, with only the matron earning 80 yuan, and nurses under twenty-five years old earned no more than 30 yuan. The younger a nurse was, the less they earned. ^30^ The income for a nurse was much lower than that of doctors running their own clinics who could earn from 300 to 3,000 yuan per month.^31^ The wage of a nurse was also lower than that of male workers in other industries. For example, in 1936, a male working in a Shanghai-based Japanese-owned textile mill could earn 55 yuan a month.^32^ A report in early 1937 reveals that the average monthly wage of workers in the printing industry reached 55 yuan. According to Gu Yongzhong (顧用中) who drew up the report, this wage was insufficient to maintain the daily life of a family of six.^33^ Male nurses faced the same difficulty. Referring again to the male nurse who had accidentally killed a patient by lethal injection, commentator Hua Xinren (花新人) stated that the man had confessed that he ran a clinic because he could not make ends meet on a nurse’s salary.^34^ Hua, who contributed several articles on nursing, did not comment on whether the salary was enough for a female nurse to support herself. It was common for women from poorer backgrounds to earn extra money to support their families in Republican China.^35^ They were not expected to be the breadwinner, and thus were less likely to be criticized by family members for their modest wages. But because male nurses did not earn a living wage, Hua concluded that they had no choice but to make their living some other way.^36^

After examining the dilemma faced by male nurses, analysts proposed several solutions to solve the problems faced by men in a nursing career. First, they proposed that male nurses be provided more educational opportunities. Generally, young men received three or four years of occupational education in nursing school after graduating middle school and were sufficiently qualified to attend college, but they still lacked the high school credential required to enter a medical college.^37^ These authors argued that this requirement of high school completion discriminated against male nurses.^38^ Tian Weifan claimed that some male nurses who had been admitted to medical college in Shanghai through personal connections, but who had not finished high school, performed outstandingly. One of Tian’s friends had taken the admission examination for Sichuan Union Medical College by showing a counterfeit high school graduation certificate. Nevertheless, he passed the exam.^39^ These examples were used to point out that male nurses had acquired enough knowledge and skill in nursing schools to further their studies in a medical college. Therefore, the commentators suggested, male nurses should be allowed to apply to medical colleges without having to finish high school.

Alternatively, commentators recommended that the state establish medical schools for public health practitioners (公醫學校 gongyi xuexiao), and open their doors to male nurses.^40^ A public health practitioner was a new position set up by the state in 1934 in county-based medical institutions. These practitioners were responsible for local issues such as the prevention of contagious diseases, research on endemic diseases, inoculation, and health care in schools. According to the document issued by the central government, only doctors who worked in clinics were qualified for this work.^41^ People who received nurses training could only work as assistants to these practitioners. Commentators believed that male nurses should be able to become doctors in public health after more education aimed directly at this position. Although men and women received essentially the same education in nursing schools, these authors did not mention that female nurses should also have access to medical schools or colleges. It appears that to them nursing was a more suitable career for women and that men were the more obvious candidates for higher education.

Suggestions for the future of nurse training reflected established gender conventions, including stereotypes of the nature of men and women and fixed domestic gender roles. One suggestion was to tailor training according to the different natures of men and women. Males were believed to work more efficiently in operating rooms, thus related courses should be available only to them.^42^ This suggestion, made by Tian Weifan, was not well received; there is no evidence to indicate that the assumption of male efficiency was put into practice in nursing schools. Another solution was to increase nurses’ salaries allowing male nurses to work in hospitals without having to find other jobs to support their families.^43^ The gender role of men as breadwinners in a family allowed them to strive for better remuneration and more job opportunities.

The employment of male nurses, however, was not as bleak as these authors imply. While the number of male nurses registered with the NAC was lower than that of female nurses in 1936, male nurses continued working and being trained in Guangxi during this period.^44^ In 1937, there were 21 male nurses registered with the police in Nanning, the capital of Guangxi province.^45^ In the same year, 185 military-trained male nurses graduated from government-run nursing schools.^46^ As there was a surplus of military nurses, the provincial government decided to dispatch them to county-based police offices and hospitals to work in public health with duties including responsibility for the purification of drinking water, hygiene of public lavatories, and inspection of drainage, households, barbers, theaters, food stores, and markets.^47^ For the government, male nurses’ work was not confined to hospitals and the military. They were welcome in various roles in improving public health. Despite the pessimism voiced by these commentators, who were mainly based in coastal cities, male nurses especially in areas such as Guangxi, were not limited to working in hospitals. Where the government had more control over the career path of nurses and competition for jobs was not as fierce, male nurses enjoyed a range of alternatives outside of low-income hospital jobs.

### Female Nurses in Civilian Hospitals: A Modern and Sexualized Image

The feminization of nursing resulted in part because male nurses sought alternative career paths. But it also came about due to hospitals trying to attract patients in a competitive marketplace. Because both private and public hospitals relied wholly or partially on patient fees for their support, they used different strategies to attract patients. Some private hospitals advertised the presence of female nurses on staff to cater to the expectation of the general population.^48^ As Barbara Mittler’s study on Chinese advertising suggests, women appeared in advertising frequently in the Republican era and were “often used as objects of male desire.”^49^ Thus it was for female nurses. Their close contact with male patients led to an erotic re-imagining of the job in popular culture. In 1937, an article in *Fengyue huabao* (風月畫報 Wind and moon magazine) stated in a mocking tone, that in a big hospital, the nurses were so sweet, pretty and modern that patients lingered in hospital even after they had recovered.^50^ Considering that the topics in this magazine generally concerned prostitutes and love affairs, this description of patients lingering because of a pretty nurse, in effect compared hospitals to brothels and implied that nursing was erotic. An article in 1939 in *Zhongguo funü* (中國婦女 Chinese women) criticized some private hospitals for featuring female nurses in their advertising to attract patients.^51^ This eroticized image of a female nurse was more frequently found in cities like Shanghai, where commercial culture flourished, and hospitals competed for clients.^52^ Women’s femininity both facilitated their access to jobs at the hospital and put them under the erotic gaze of the public in a commercial society.

In public hospitals run by provincial governments, female nurses were represented as New Women to support the idea of the modernity of the hospitals. The trope of the New Woman was a cultural symbol that encompassed the ideal of the modern female citizen. Two features of female nurses were highlighted: their professionalism and their presence in the public sphere. The white gowns they wore signaled professionalism, attire that distinguished them as specialists who “possessed the authority of medical knowledge,” acquired through a Western-style education.^53^ Receiving an education was an expectation required of the New Woman after the May Fourth Movement and nurses, fresh out of nursing school, were ideal models of the New Woman in this sense. Second, female nurses were contrasted with the traditional woman confined to the “inner chamber.” Working in the public sphere, they had contact with people from all walks of life. Thus, hospitals that hired female nurses could utilize the New Woman image to popularize the modern medical services they provided. By 1934, photos from hospitals published in medical magazines, were frequently of women working in wards, surgery rooms, and outpatient rooms, while men were rarely shown, though it was the male doctors who treated the patients and enjoyed higher status.^54^ Identifying female nurses as the New Woman upheld the ideal of female citizenship: the women were modern because of their work. And the New Woman exhibited virtues of seriousness and frugality, in contrast to the Modern Girl who preferred foreign goods and indulged in vacuous entertainment. Governments at different levels, aiming to prevent moral degradation caused by the underbelly of modernity and crass commercialism, also welcomed female nurses’ identity as the New Woman. Photos from Wuzhou Provincial Hospital, for example, published in *Guangxi Weisheng xunkan* (廣西衛生旬刊 Guangxi hygiene periodical) in 1934, adopted the image of the New Woman as an advertising strategy. In all the photos featuring medical professionals, readers see female nurses working in their uniforms. Male doctors can only be found in two photos, and there are no photos of male nurses (Figure 1, 2, 3, 4 and 5).^55^ Even in state-run hospitals women were preferred when it came to advertising as they connoted modernity and the ideal of the female citizen.

Even though female nurses were often depicted as the New Woman, they did not entirely escape suspicion that they were really the Modern Girl. The very femininity they displayed by virtue of their modern appearance made them suspect. Inpatient Xu Ruiling (徐蕊玲) complained that female nurses took time with their hairstyles and makeup and wore fashionable footwear because shoes were not a regulated part of the uniform. Xu said they wore the uniform simply to be “dressed like a nurse” (要裝得像一個護士小姐).^56^ Xu’s description reminded readers of the Modern Girl in advertisements who dressed in stylish ways and consumed foreign goods. The charming appearance of female nurses in advertisements contrasted with the serious and professional figure of nurses that missionaries, the government, and the NAC had tried to create by standardizing the uniform. The fashionable image implied that female nurses were slacking off in their work and that made the public uncomfortable. This concern about female nurses’ appearance was not only found in Shanghai. The Guangxi Military Hospital issued a regulation in April 1937 requiring female nurses and pupils to bind their hair during work and to forego having their hair curled. They were also not allowed to use make-up or wear stylish clothes, even when they were on their own time.^57^ These regulations on hairstyle, make-up and clothing during leisure time reveal that any resemblance to the Modern Girl displayed by a female nurse was a problem in the mind of the Guangxi Government.

For some commentators and patients, female nurses took on attributes of the Modern Girl because their appearance represented a display of their vanity. According to the writer Xu Ruiling, female nurses spoke English to her when discussing her symptoms. When Xu Ruiling answered them in Chinese, she felt they looked down on her as a country bumpkin. If Xu spoke in English, nurses either put on a poker face, told her to speak Chinese or ignored her.^58^ For Xu, speaking English meant the nurses appreciated the foreign language more than Chinese. It was too reminiscent of the preference for foreign goods over domestic ones, something that the Nationalist Government denounced. Though Xu’s criticism might have exaggerated the vanity of female nurses, it nevertheless revealed patients’ skepticism of modern traits shown by the nurses. Ultimately, the modernity of these female nurses was deemed superficial when they were unable to have a full English conversation with the patient. Their attempts at English echoed the idea of the Modern Girl as one who received only a smattering of education and who remained unaware of her own inability. She may have worked in an urban setting, led a modern lifestyle, and tried to climb the social ladder, but she remained bound by her lower standing in society.^59^ The supposed vanity of female nurses stood in contradiction to the virtues of frugality and patriotism advocated by the Nationalist Government, especially after the New Life Movement in 1934.

Some thought that the modern traits of female nurses posed a threat to feminine virtues promoted at the time. Xu and another writer, Yinuo (以諾), complained that nurses chatted at work about the activities they would do during their time off, such as going to the movies and swimming.^60^ These authors believed that discussing popular activities while at work damaged the professional image of female nurses.^61^ An even more serious problem described by the writers was the nurses’ flirting with doctors during work and talking about dating.^62^ Female nurses were not prohibited from dating, but Xu’s complaint reveals that talking about relationships was improper. Pursuing love freely was another trait of the New Woman, who had previously been consigned to arranged marriages. Women discussing dating and flirting with men, however, fell under the trope of the Modern Girl with her dreams of a better life through marriage and who did not take her work seriously. Without contemporaneous notes of the conversations between the nurses and the doctor, it is unclear whether the nurses flirted or simply chatted. No record exists to show whether they talked to a male doctor about subjects outside of the realm of their professions. Still, this type of behavior was considered flirting, and society saw it as a threat to social norms about chastity. Female nurses who challenged the feminine virtues such as chastity and frugality, often upheld by intellectuals and political elites, became the target of their middle-class clients. These criticisms implied that an increasing number of women were in the workforce as nurses, and that caused anxiety among the middle-class citizens worried about the old moral codes.

Along the same lines, middle-class patients also worried about the similarity between nursing and maids’ work. In 1936, Chen Zhu Bihui, Dean of the Central Senior Nursing School in Nanjing and the first chairwoman of the Shanghai NAC, criticized those who held the notion that nurses served as maids of patients and attendants of doctors.^63^ She considered such views as biased, coming from those unfamiliar with the occupation of nursing. Before missionaries introduced nursing to China, there was no profession that took care of patients; they were either cared for by servants or their family members. Even though it was common to employ nurses in civilian hospitals by the mid-1930s, the populace generally did not recognize nurses’ professionalism. From a patients’ perspective, nurses made their beds, took their temperature, fetched medicine, and removed bedpans, none of which required medical knowledge.^64^ To the wealthy, such work was done by maids, and they weren’t ready to distinguish between nurses and maids, except to recognize that nurses worked in hospitals and were not the employee of the patient. This view discounted the diverse class backgrounds found among nurses. While nursing was deemed a working-class profession, not all female nurses in China were from a poor background nor a working-class family. In fact, the training program provided by PUMC attracted pupils from the better-off classes.^65^ Due to the similarities between nursing and maids’ work, however, in the mind of the middle-class patient, female nurses, regardless of their backgrounds, were essentially maids in hospitals. Chinese nurses from the same class as their critics were more likely to be scapegoated and blamed by middle-class people who felt that well-established feminine virtues and moral codes were being challenged by a new culture and ideas.^66^ Class snobbery conflated with gender stereotypes, making female nurses prey to those who feared the collapse of conventional feminine virtues and moral codes.

Criticism against female nurses reinforced existing gender stereotypes, which were relatively invisible because they were so prevalent.^67^ Commentators and patients took it for granted that women should be gentle and take good care of patients. When nurses who occupied a leading position in government-run hospitals and organizations set up ethical rules for the profession, they automatically declared that the care of patients required nurses to be considerate and mild to patients. In the mind of the patients, however, male nurses stood apart and did not have to follow the same ethical norms. Reflections on the work of male nurses did not refer to their performance in a hospital, though many such musings mentioned that the male temper made them less suitable for nursing. Yinuo, a critic of both male and female nurses’ performances, nevertheless only empathized with men’s busy work schedule saying it explained their poor attitudes towards patients.^68^ This kind of expectation for female and male nurses was based on perceived gender differences, thus strengthening the idea that women should display “inherent” feminine virtues of kindness and gentleness. Meanwhile, patients who complained about female nurses’ mistakes attributed the errors to the deficiencies of the female sex! When Yinuo noted female nurses’ conversations about their dating lives, she explained that women were prone to romantic notions which was why they talked about it during work time. By contrast, men were seen as keen to earn money to support their family, without any spare time for pursuing love.^69^ The comparison between sentimental women and sensible men suggested that women’s innate feminine traits were the root cause of their poor attitude in the workplace, stigmatizing them as unsuitable for serious working environments. But just as criticism of “feminine” traits tarnished female nurses’ professional image, it also provided them with a path forward to justify their role in the nursing profession.

### Nursing as a Profession for Women: Professionalization and Feminization

Faced with these criticisms, female nurses emphasized the high moral standards in nursing that distinguished it from maids’ work, and they rejected the idea that women were too emotional to focus on their work. Chen Zhu Bihui discussed the tolerance and sympathy practiced by nurses, saying that because patients were more peevish than healthy people, nurses needed to empathize; nurses had to intimately understand the bad temper of patients. “Being empathetic,” she explained, “means that ‘you should not impose on others what you yourself do not desire’ (己所不欲, 勿施於人 *jisuobuyu wushiyuren*).”^70^ This variant of the Golden Rule was cited from *The Analects of Confucius* (論語 *Lunyu*) and was well-known to virtuous men. By making these two points, Chen Zhu depicted nurses as having higher moral standards, whether they were born to it or educated in this path. Nurses worked because of their benevolence and thus were different from maids who passively obeyed the orders of their masters. Chen Zhu argued that patients should respect nurses rather than treat them in a master/servant relationship. This discussion also implied that being sentimental was an asset to a nurse; female nurses could offer more to their patients than their male counterparts.

Another way to establish a professional identity for female nurses was to showcase the skills and knowledge required for nursing. Chen Zhu informed her readers that a qualified nurse had to have a command of middle school knowledge before they received a medical school education.^71^ She then listed the compulsory courses required to complete nursing school. Even as she introduced nursing training to the public and opened the discussion of a suitable plan for nurse trainees, her words inevitably suggested that the rigor of nursing was not for the average person.^72^ In fact, Chen Zhu’s article became an example to show how well-educated a nurse could be. Her elegant writing style, use of proverbs, command of Latin, and understanding of why and how nursing should be developed demonstrated her own impressive educational background.^73^

Although femininity was a reason why female nurses were scrutinized by patients more strictly than men, nurses did not shun gender discourse during their work to speed the professionalization of their work. While female nurses’ self-identification and practice in the workplace were inevitably shaped by public opinion about womanhood, they consciously avoided any weakness that was generally attributed to women.^74^ Instead, they picked the positive characteristics associated with womanhood and motherhood to establish their status on the wards. For example, nurses compared the relationship between themselves and their patients to the relationship between mother and child. Chen Zhu referred to the etymology of the term “nurse” to illustrate what relationship a nurse should have with her patients. In Latin “to nurse” means to take care of others. Chen Zhu, though not the first to make this analogy, said that nurses must take care of patients just as mothers rear their children. In 1925, an article in *Guangji yikan* (廣濟醫刊 Guangji medical journal) drew the same conclusion while discussing the responsibility of nurses. The article further claimed that since nurses resembled “mothers of patients in wards,” the sick should be obedient to the nurses who held a position of higher status.^75^ Nurses tolerated the bad tempers of patients, not because they were maids, but because they understood they were the ones with medical knowledge, and that gave them authority over patients’ activities. Nurses decided what was allowed in the ward and they prevented patients from inappropriate actions. This discourse emphasized the power relationship between nurses and patients and did not touch on the actual activities involved in taking care of patients. This new nurse/patient relationship dynamic reflected an ideal envisioned by the medical professional where patients were fully obedient to doctors and nurses. According to Yin, however, such a dynamic was not achieved in the first half of the twentieth century,^76^ and it is likely that the nurses’ power over patients remained largely on paper. In reality, they struggled to earn their patients’ respect. This aspirational change in nurses’ status, began to distinguish the professional nurse from the servant and it also deflected any patient criticism because now the patient was often seen as a sulky child whose words could not be taken seriously.

Along with reworking the power dynamic between patients and nurses, nurses also drew attention to the hierarchy between doctors and nurses. Chris Nottingham has observed that nurses in nineteenth-century England were insecure professionals who may have been recognized by education and certification, but who were still not perceived as having an “authoritative social performance.”^77^ The same held true in China. Although people understood that nurses underwent years of formal training, they considered nurses the assistants of doctors. The hierarchy was most obvious where doctors ordered nurses to do things beyond their assigned duty and outside their professional sphere. *Nüzi yuekan* (女子月刊 Monthly journal of women), for example, published several paragraphs from a nurse’s diary in 1937. In one excerpt, the nurse complained that she had had to mend a doctor’s socks.^78^ It is possible this article is fictional. But it delivers enough truth on the lower status of nurses, that the public could well have read it as if it were real.

Nurses were keen to reject the identity of being doctors’ assistants. One of the tactics they used to avoid being viewed as less knowledgeable than doctors was to differentiate their responsibilities from those of doctors. Qi Yun (琪雲), a nurse who published two articles in *Minli zhoukan* (民力週刊 Weekly journal of people’s power), wrote that nurses took care of patients as a complement to doctors’ treatment. They were not subordinate to doctors but held an equally important role in a hospital.^79^ In an interview in 1943, a nurse named Xiong Wenying (熊文英) talked about her experience taking care of a typhus patient. According to Xiong, the patient was in poor condition when admitted into the hospital. “People thought the patient was doomed” (大家都覺得他是沒有多大希望了). She helped him bathe and cleaned his back every day, she provided medicine, managed his meals, and carried out the doctor’s recommendations. The patient got better and was able to walk after two months. The nurse understood that the patient’s recovery was dependent on the medicine prescribed, but she also knew that without her care, the patient might have suffered complications, such as pneumonia.^80^ Some nurses even went so far as to argue that they should be allowed to intervene in treatment. In 1947, Guan Baozhen (管葆真 1908-1993), a member of the board of the NAC, suggested that doctors should discuss the treatment of patients with nurses and even help nurses in their work.^81^ Her advice underlined the idea that doctors and nurses in hospitals should share the equal status of healthcare provider. Although this ideal scenario was never achieved, Guan Baozhen’s words reveal an ambitious goal: expand the role of nurses and improve their status in the hospital.

By professionalizing nursing, female nurses also accelerated the feminization of the occupation. In encouraging women to take up nursing, advocates underscored both the professionalism of nursing and how well nursing suited women’s traditional gender roles. Chen Zhu called nursing the best job for women. She stressed that women could gain an education and find a job, essential elements if women were to break out of the Confucian conventions that confined their activities to the home. Women’s education and economic independence were the basis for gender equality. Somewhat incongruously, Chen Zhu also concluded that women would excel in their traditional gender roles at home after marriage, if they had worked as a nurse. ^82^

This opinion echoed the emphasis on domestic science that had begun in the late 1920s. Women were encouraged to study home economics to prepare themselves to be wives who could manage a household with scientific skill. Intellectuals and politicians considered it important for women to successfully manage the domestic space because their work improved the health and well-being of the nation.^83^ This line of thinking viewed women who received training as nurses, as better equipped to take care of their family members in a scientific way. Thus, nursing programs provided women with transferrable skills for both a profession and their domestic role. Their work was important to the nation; they saved lives as nurses, and reared healthy citizens. Chen Zhu’s analysis of the importance of female nurses revealed a paradoxical view of working women. A woman was expected to be economically independent, and her equality to men was demonstrated by her education and work. Yet, she was still bound by social norms to become a wife and mother. Her education and work experience became even more valuable when she could apply them to managing her household.

## Conclusion

From the 1920s to the 1940s, nursing was discursively constructed as a woman’s occupation. By the 1930s nursing had ceased to be a good career option for men and was now considered an occupation suitable only for women. Men who had trained as nurses in the early 1920s when they could slip the bounds and perform doctor’s work, were barred from this practice due to the stricter regulation of medical practices by the end of the decade. Moreover, men themselves were dissatisfied with the low salaries afforded nurses, considering them insufficient to support a family. Male nurses sought other jobs to earn more money. Meanwhile, both commentators and the general population believed that female nurses were more suitable in hospitals for civilians because they were gentler and more adept at taking care of patients. The work of nurses was an expansion of women’s role at home. Although there were suggestions to increase nurses’ pay and provide more opportunities, no evidence suggests that the government or the NAC took any action in this direction.

Popular images of female nurses both boosted and undermined women’s competitiveness in nursing compared with their male counterparts. Civilian hospitals preferred female nurses because their modern education and professionalism upheld the New Woman ideal. Yet, despite the modernity they represented, female nurses were criticized for the same traits that reminded people of the stylish, materialistic, and vain Modern Girl…younger sister of the New Women. They were disparaged because they exposed their femininity to the public and violated the popular understanding of feminine virtue. Female nurses were not criticized directly for their intimate contact with patients, but the violation of gender boundaries still led to an erotic reimagining of the nurse’s work. Patients scrutinized the work of female nurses and attributed the perceived lack of professionalism to feminine characteristics, while male nurses did not face the same criticisms. Middle-class clients often had a skeptical attitude toward female nurses because the nurses were from the working class. Class snobbery and gender bias scapegoated those women who engaged in a working-class profession that seemed to challenge traditional moral codes and female virtues.

Female nurses were able to use their education and popular opinion about gender roles to counter the criticisms caused by gender bias. To professionalize nursing, they promoted the feminization of the profession. They emphasized the knowledge and virtues required of a good nurse and took pains to distinguish nurses from maids and to deny that women were too sentimental to be good nurses. They also made an analogy between the nurse-patient relationship and that between mother and child to establish a favorable power dynamic. This hierarchy, however, could only work when patients recognized the authority of nurses, which they were not able to do in the Republican era. Nurses sought to achieve an equal status with doctors in the hospital, going counter to the notion that they merely assisted them. Female nurses encouraged more women to choose nursing, declaring that it allowed them to become New Women with skills that could transfer to being a good wife and mother. Ultimately, being a good wife and mother were upheld as the gendered responsibilities of female citizens in the Republican era.

The outbreak of the Second Sino-Japanese War provided female nurses with a wider range of opportunities, including in military nursing. This again accelerated the professionalization and feminization of nursing. During this period labor shortages allowed women access to jobs on the lower rungs of the medical profession. The experience of Zhou Meiyu (周美玉 1910-2001), future Lieutenant General in the Republic of China, suggests that individual female nurses could ascend to even higher status in medical institutions and government departments. Although the war’s influence on nursing is beyond the scope of this article, the Second Sino-Japanese War constitutes another important phase in the development of nursing and in the overall history of women working outside the home in Republican China.

## Footnotes

1 Xiao Linlin, “Shanghai hushi qunti yanjiu (1927-1937)” [Nurses as a professional cohort in Shanghai (19277-1937)] (MA thesis, Shanghai Normal University, 2022), 36.

2 “New Members of the Nurses’ Association of China,” *Nursing Journal of China* 17, no. 4 (1936): 262.

3 Shanghaishi difangzhi bianzuan weiyuanhui [Editors’ Committee of Shanghai Gazetteers], *Shanghai shiji zhuanzhi Renji yuyuan zhi* [Shanghai Municipal Gazetteers: Renji Hospital] (Shanghai: Shanghai Scientific and Technological Literature Press, 2019), 187.

4 Guangxi Wuzhoushi gongren yiyuan, *Wuzhoushi gongren yiyuan zhi* [Gazetteer of the workers’ hospital in Wuzhou] (Hong Kong: Xianggang tianma tushu youxian gongsi, 2004), 214.

5 “N.A.C. Historical Summary,” *Quarterly Journal for Chinese Nurses* 8, no. 3 (1927): 15.

6 “Guomin zhengfu weishengbu zuzhifa,” [The plan of establishment of the Ministry of Public Health Administration of the Nationalist Government], *Xingzhengyuan gongbao* [Gazette of Executive Yuan] 3 (1928): 6 -10.

7 Sarah E. Stevens, “Figuring Modernity: The New Woman and the Modern Girl in Republican China,” *NWSA Journal* 15, no. 3 (2003): 85-88.

8 Louise Edwards, “Policing the Modern Woman in Republican China,” *Modern China* 26, no.2 (2000): 132-7. Gail Hershatter, *Women and China’s Revolutions* (Lanham, MD: Rowman & Littlefield, 2019), 149.

9 Yen Hsiao-pei, “Body Politics, Modernity and National Salvation: The Modern Girl and the New Life Movement,” *Asian Studies Review* 29 (2005): 166.

10 Hershatter, *Women and China’s Revolutions*, 143.

11 Julia Hallam, *Nursing the Image: Media, Culture and Professional Identity* (London: Routledge, 2000), 20; Elizabeth Lunbeck, *The Psychiatric Persuasion: Knowledge, Gender, and Power in Modern America* (Princeton, N.J: Princeton University Press, 1994), 26.

12 Gail Hershatter, *The Workers of Tianjin, 1900-1949* (Stanford, CA: Stanford University Press, 1986); Emily Honig, *Sisters and Strangers: Women in the Shanghai Cotton Mills, 1919-1949* (Stanford, CA: Stanford University, 1986); Jacob Eyferth, “Women’s Work and the Politics of Homespun in Socialist China, 1949–1980,” *International Review of Social History* 57, no. 3 (2012): 365-91; Angelina Chin, *Bound to Emancipate: Working Women and Urban Citizenship in Early Twentieth-Century China and Hongkong* (Plymouth, UK: Rowan & Littlefield Publishers, 2012); Nicole Elizabeth Barnes, *Intimate Communities: Wartime Healthcare and the Birth of Modern China, 1937–1945* (Oakland, CA: University of California Press, 2018); Joshua H. Howard, “The Politicization of Women Workers at War: Labor in Chongqing’s Cotton Mills during the Anti-Japanese War, *Modern Asian Studies* 47, no. 6 (2013): 1888-1940; S.A. Smith, *Revolution and the People in Russia and China: A Comparative History* (Cambridge: Cambridge University Press, 2008), 111-50.

13 Hershatter, *The Workers of Tianjin*; Honig, *Sisters and Strangers*.

14 Gail Hershatter, *The Gender of Memory Rural Women and China’s Collective Past* (Berkeley: University of California Press, 2011).

15 Shenglan Li, “A Social History of Wartime Nursing Training in Hunan, 1937-1945,” in *China and the Globalization of Biomedicine*, ed. David Luesink et. al. (New York: University of Rochester Press, 2019), 159-182; Sonya Grympa and Cheng Zhen, “The Development of Modern Nursing in China,” in *Medical Transitions in Twentieth Century*, ed. Bridie Andrews and Mary Brown Bullock (Bloomington, IN: Indiana University Press, 2014); Chen Xing, “Tizhi jiangou yu linian chuanbo: Zhonghua huli xuehui yanjiu, 1909–1949” [System construction and spread: The study on Nurses’ Association of China, 1909–1949] (MA thesis., Hebei University, 2014); Sonya Grympa, *Nursing Shifts in Sichuan: Canadian Missions and Wartime China, 1937–51* (Vancouver: University of British Columbia Press, 2021); Barnes, *Intimate Communities*, 52-90. For the backlash on women’s working in public, see Howard, “The Politicization of Women Workers at War: Labor in Chongqing’s Cotton Mills during the Anti-Japanese War,” 1902-3.

16 Jiang, “Shaping modern nursing development in China before 1949,” 22.

17 Chen, “Tizhi jiangou yu linian chuanbo,” 54.

18 Chen, “Tizhi jiangou yu linian chuanbo,” 56-7.

19 Yin Qian, “Minguo shiqi de yishi qunti yanjiu (1912–1937): yi Shanghai wei zhongxin” [Study on medical practitioners in Republican China (1912–1937): with Shanghai as a focus] (PhD diss., Central China Normal University, 2008), 2.

20 Cai Hongyuan, ed., “Yishi zanxing tiaoli” [Temporary ordinance of doctors], *Minguo fagui jicheng* [Collection of laws in the Republican era] 40 (Hefei: Huangshan chubanshe, 1999), 257.

21 “Shengdian yixueyuan deng guanyu qudi weiling yingyezhizhao zhi xiyi deng yian yang ge fenbie zunban you” [Telegram from the provincial government to the medical college on clamping down on practicing Western medicine without license], *Guangxi shengzhengfu gongbao* [Guangxi provincial government gazette] 68 (1935): 35–36.

22 Yin, “Minguo shiqi de yishi qunti yanjiu (1912–1937), 79.

23 Tian Weifan, “Guanyu nanhushi chulu zhi shangque” [Discussion on the future of male nurses], *Yishi gonglun* [Public opinions on medicine] 2, no. 19 (1935): 9; Xie Bingshi, “Zhongguo hushi jiang wang nali qu” [What is the future of the male nurses in China], *Yishi gonglun* [Public opinions on medicine] 2, no. 18 (1935): 19.

24 Hsianglin Lei, *Neither Donkey nor Horse: Medicine in the Struggle Over China’s Modernity* (Chicago: The University of Chicago Press, 2014).

25 Hua Xinren, “Nanhushimen dangqian yanzhong de wenti: Dule maopai yishi dazhen sharen xinwen hou” [A major problem concerning male nurses: After reading the news on a fake doctor administering a lethal injection], *Yishi gonglun* [Public opinions on medicine] 4, no. 43 (1936): 23.

26 Charlotte Bacon, “Changing Opportunities in Kwangsi,” August 1935, pp 37, file CMZ/M/C 2/1 Cadbury Research Library, University of Birmingham.

27 Bacon, “Changing Opportunities in Kwangsi,” pp 37.

28 Tian, “Guanyu nanhushi chulu zhi shangque,” 8.

29 Hua, “Nanhushimen dangqian yanzhong de wenti,” 23–5.

30 Xiao, “Shanghai hushi qunti yanjiu (1927-1937), 73.

31 Xu Silin, “Minguoshiqi Shanghai nüxiyi yanjiu” [Female doctors in Republican Shanghai] (MA thesis, Jinan University, 2020), 71.

32 Zhang Chi, “Jindai Qingdao fangzhi gongren yanjiu (1914-1937)” [A study on textile workers in Qingdao (1914-1937)] (MA thesis., Qingdao University, 2022), 46.

33 Gu Yongzhong, “Shanghai bagong de yiban zhuangkuang (fu tubiao)” [The living condition of printing workers in Shanghai (with forms)], *Xinwen jizhe* [Reporters] 1, no. 2: 12-6.

34 Hua, “Nanhushimen dangqian yanzhong de wenti,” 23–5.

35 Weikung Cheng, *City of Working Women: Life, Space and Social Control in Early Twentieth-Century Beijing* (Berkeley: University of California, Berkeley, 2011).

36 Hua, “Nanhushimen dangqian yanzhong de wenti,” 24.

37 “Guangxi shengli yiyuan benke, zhuanxiuke ji fushe hushizhuchanban zhaosheng guanggao” [Admission advertisement of Guangxi provincial medical college, medical school and the project of nursing and midwifery training], *Nanning minguo ribao* [Nanning republican daily], August 23, 1935, 7.

38 Xie, “Zhongguo hushi jiang wang nali qu,” 20; Tian, “Guanyu nanhushi chulu zhi shangque,” 10.

39 Tian, “Guanyu nanhushi chulu zhi shangque,” 9.

40 Xie, “Zhongguo hushi jiang wang nali qu,” 20; Tian, “Guanyu nanhushi chulu zhi shangque,” 9–10.

41 Zang Shiyi and Bao Kang, “Wei zhiding gongyi guize gongbu you” [For showcasing how to draft regulations on public health practitioners], *Minzhengbu banyuekan* [Journal of Ministry of the Interior half-monthly] 1, no. 4 (1934): 4.

42 Tian, “Guanyu nanhushi chulu zhi shangque,” 10.

43 Hua, “Nanhushimen dangqian yanzhong de wenti,” 25.

44 “New Members of the Nurses’ Association of China,” 262.

45 “Nianwunianfen benshi yiyao zhuangkuang” [The state of medicine in this city in 1937], *Nanning minguo ribao* [Nanning republican daily], March 6, 1937, 7.

46 Nurses who received training provided by government-run institutions in Guangxi were required to work within the province as the government designated for a fixed time.

47 Liu Yuan, “Minguo shiqi Guangxi qingjie weisheng gongzuo gaikuang” [Summary on hygiene and public health in Guangxi during the Republican Era], *Guangxi yiliao weishengzhi shiliao tongxun* [Collection of primary sources on medicine and public health in Guangxi] 12 (1992): 38.

48 Xie, “Zhongguo hushi jiang wang nali qu,” 19.

49 Barbara Mittler, “Gendered Advertising in China: What History do Images Tell,” *European Journal of East Asian Studies* 6, no.1 (2007): 22–3.

50 *Wind and moon* (風月 *fengyue*) connotes brothel or amorous women in Chinese. Liuhuo, “Rulangsihu nühushi” [Fierce and erotic female nurse], *Fengyue zazhi* [Wind and moon magazine] 10, no. 29 (1937): 1.

51 Hezhen, “Guanyu hushi de jijuhua” [Some words on female nurses], *Zhongguo funü* [Chinese women] 1, no. 10 (1939): 23.

52 For example, *Fengyue huabao* was a Shanghai-based magazine. The hospital criticized by *Zhongguo funü* was also in Shanghai.

53 Barnes, *Intimate Communities*, 132.

54 See *Guangxi weisheng xunkan* [Guangxi hygiene periodical] 2, no. 1 (1934): 33–34, accessed November 27, 2023, https://www.cnbksy.com/home.

55 *Guangxi weisheng xunkan*, 33–34.

56 Xu Ruiling, “Hushi xiaojie de fuwu jingshen” [The spirit of service among female nurses], *Funüjie* [The women] 9 (1940): 13.

57 “Guangxi junyiyuan qudi hushi xishang fuhua” [Military Hospital in Guangxi banned nurses’ flashy lifestyle], *Jianshe yixue yuekan* [Medical monthly for a Health Society] 2, no. 9 (1937): 94.

58 Xu, “Hushi xiaojie de fuwu jingshen,” 13.

59 Tze-lan D. Sang, “The Modern Girl in Modern Chinese Literature,” in *A Companion to Modern Chinese Literature*, ed. Yingjin Zhang (West Sussex: John Wiley & Sons, 2016), 411-23.

60 Yinuo published an article in *Nüduo* (女鐸 A woman’s messenger) a magazine created by the Methodist Church in China to promote the liberation of women.

61 Xu, “Hushi xiaojie de fuwu jingshen,” 13. Yinuo, “Bingren yinxiangzhong de nühushi” [Female nurses in the patient’s mind], *Nüduo* [A woman’s messenger] 29, no. 4 (1940): 29–30.

62 Xu, “Hushi xiaojie de fuwu jingshen,” 13; Yinuo, “Bingren yinxiangzhong de nühushi,” 29–30.

63 Chen Zhu Bihui, “Tantan woguo hushi zhiye” [On the profession of nursing in China], *Guangbo zhoubao* [Weekly journal of broadcasting] 70 (1936): 43.

64 Guo Renji, “Lun wuguoren ying juyou zunzhong *nü*zi hushi zhiye zhi guannian” [On that our people should respect female nurses], *Shehui yiyao* [Social medicine] 3, no. 12 (1936): 3.

65 Grympa, *Nursing Shifts in Sichuan*, 37

66 Lunbeck, “‘A New Generation of Women,’” 513-43.

67 Lunbeck, *The Psychiatric Persuasion*, 26.

68 Yinuo, “Bingren yinxiangzhong de nühushi,” 30.

69 Yinuo, “Bingren yinxiangzhong de nühushi,” 30.

70 Chen Zhu Bihui, “Tantan woguo hushi zhiye (xu)” [On the profession of nursing in China (part two)], *Guangbo zhoubao* [Weekly journal of broadcasting] 71 (1936): 39.

71 Chen Zhu, “Tantan woguo hushi zhiye (xu),” 40.

72 Chen Zhu, “Tantan woguo hushi zhiye (xu),” 40.

73 Chen Zhu Bihui graduated from the Senior Nursing School of PUMC (協和高級護校 Xiehe gaoji huxiao) in 1925.

74 For women’s self-identification in the work, see Lunbeck, *The Psychiatric Persuasion*, 26; Elizabeth Lunbeck, “‘A New Generation of Women’: Progressive Psychiatrists and the Hypersexual Women,” *Feminist Studies* 13, no. 13 (1987): 513-43.

75 Qi Ao, “Lun zenyang raokecheng hege de hushi” [On how to be a qualified nurse], *Guangji yikan* [Guangji medical journal] 2, no. 1 (1925): 23.

76 Yin, “Minguo shiqi de yishi qunti yanjiu,” 212.

77 Chris Nottingham, “The Rise of the Insecure Professionals,” *International Review of Social History* 52, no. 3 (2007): 430.

78 Baihe, “Kanhufu riji chao” [Paragraphs from the diary of a nurse], *Nüzi yuekan* [Monthly journal of women] 5, no. 7 (1937): 29–31.

79 Qiyun, “Hushi zai funü zhiyejiede zhongyaoxing” [The importance of nursing among all professions for women], *Minli zhoukan* [Weekly journal of people’s power] 11 (1939): 17.

80 “Hushi zuotanhui” [Symposium for nurses], *Funü zazhi* [Journal of women] 4, no. 5 (1943): 25.

81 Guan Baozhen, “Gei yishimen de yifeng gongkaixin” [A public letter to doctors], *Zhongguo hushi jikan* [Quarterly journal for Chinese nurses] 1, no. 2 (1947): 3–4.

82 Chen Zhu, “Tantan woguo hushi zhiye,” 43.

83 Helen M. Schneider, *Keeping the Nation’s House: Domestic Management and the Making of Modern China* (Vancouver: UBC Press, 2008), 1-2.

